# Poly(ADP-Ribose)Polymerase (PARP) Inhibitors and Radiation Therapy

**DOI:** 10.3389/fphar.2020.00170

**Published:** 2020-03-03

**Authors:** Stephen A. Jannetti, Brian M. Zeglis, Michael R. Zalutsky, Thomas Reiner

**Affiliations:** ^1^ Department of Biochemistry, Hunter College, New York, NY, United States; ^2^ Ph.D. Program in Biochemistry, CUNY Graduate Center, New York, NY, United States; ^3^ Department of Radiology, Memorial Sloan Kettering Cancer Center, New York, NY, United States; ^4^ Ph.D. Program in Chemistry, CUNY Graduate Center, New York, NY, United States; ^5^ Department of Radiology, Duke University Medical Center, Durham, NC, United States; ^6^ Department of Radiology, Weill Cornell Medical College, New York, NY, United States; ^7^ Chemical Biology Program, Memorial Sloan Kettering Cancer Center, New York, NY, United States

**Keywords:** PARP [poly(ADP-ribose) polymerase], radiotheranostic, molecular imaging, targeted radiotherapy (TRT), combination therapy

## Abstract

Poly(ADP-ribose)polymerase-1 (PARP1) is a DNA repair enzyme highly expressed in the nuclei of mammalian cells, with a structure and function that have attracted interest since its discovery. PARP inhibitors, moreover, can be used to induce synthetic lethality in cells where the homologous recombination (HR) pathway is deficient. Several small molecule PARP inhibitors have been approved by the FDA for multiple cancers bearing this deficiency These PARP inhibitors also act as radiosensitizing agents by delaying single strand break (SSB) repair and causing subsequent double strand break (DSB) generation, a concept that has been leveraged in various preclinical models of combination therapy with PARP inhibitors and ionizing radiation. Researchers have determined the efficacy of various PARP inhibitors at sub-cytotoxic concentrations in radiosensitizing multiple human cancer cell lines to ionizing radiation. Furthermore, several groups have begun evaluating combination therapy strategies in mouse models of cancer, and a fluorescent imaging agent that allows for subcellular imaging in real time has been developed from a PARP inhibitor scaffold. Other PARP inhibitor scaffolds have been radiolabeled to create PET imaging agents, some of which have also entered clinical trials. Most recently, these highly targeted small molecules have been radiolabeled with therapeutic isotopes to create radiotherapeutics and radiotheranostics in cancers whose primary interventions are surgical resection and whole-body radiotherapy. In this review we discuss the utilization of these small molecules in combination therapies and in scaffolds for imaging agents, radiotherapeutics, and radiotheranostics. Development of these radiolabeled PARP inhibitors has presented promising results for new interventions in the fight against some of the most intractable cancers.

## Introduction

Poly(ADP-ribose)polymerase-1 (PARP1) is a 116 kDa DNA repair enzyme with nuclear concentrations ranging from 2 × 10^5^ to 1 × 10^6^ enzymes/nucleus in eukaryotic cells (Ludwig et al., 1988; Herceg and Wang, 2001). Within 30 seconds of the advent of DNA damage, PARP PARylates itself, activating the enzyme and leading to a 500-fold increase in its activity over basal levels (Benjamin and Gill, 1980; Alvarez-Gonzalez and Althaus, 1989; Haince et al., 2008; Hassa and Hottiger, 2008; Langelier et al., 2012). Unsuccessful PARP1 mediated repair can result in cell death through multiple pathways, including apoptosis (Kaufmann et al., 1993), ATP depletion (Martin et al., 2000), parthanatos (David et al., 2009), and mitotic catastrophe (Schoonen and van Vugt, 2018).

Over the past decade, inhibitors of PARP have emerged as a common monotherapy for certain subtypes of breast and ovarian cancers (Tangutoori et al., 2015). Moreover, preclinical data has demonstrated that PARP inhibition can increase radiosensitivity in cancer cells (Wang et al., 2019). The efficacy of combination therapies employing PARP inhibitors and external beam radiation has been demonstrated in the clinic, and several phase I clinical trials based on this approach have been completed at the time of writing (NCT00770471, NCT00649207, NCT01264432, NCT01477489, NCT01514201, NCT01657799), with results being available for some of them (Russo et al., 2009; Tangutoori et al., 2015; Dréan et al., 2016). The use of PARP inhibitors as scaffolds for radiopharmaceuticals has also blossomed in recent years (Irwin et al., 2014; Salinas et al., 2015; Carney et al., 2016; Carney et al., 2017; Jannetti et al., 2018; Reilly et al., 2018; Makvandi et al., 2019; Pirovano et al., 2019). To wit, several clinical trials of PARP-inhibitor-based diagnostic imaging agents are currently in progress or have been completed ([^18^F]FluorThanatrace (Michel et al., 2017), PARPi-FL (Kossatz et al., 2019)), and [^18^F]PARPi (Schöder et al., 2019)) and a number of therapeutic radiopharmaceuticals based on PARP inhibitors have been employed in preclinical animal models (Kossatz et al., 2016; Michel et al., 2017; Sander Effron et al., 2017).

## Mechanism of PARP Inhibition

### DNA Binding

**Figure 1:**
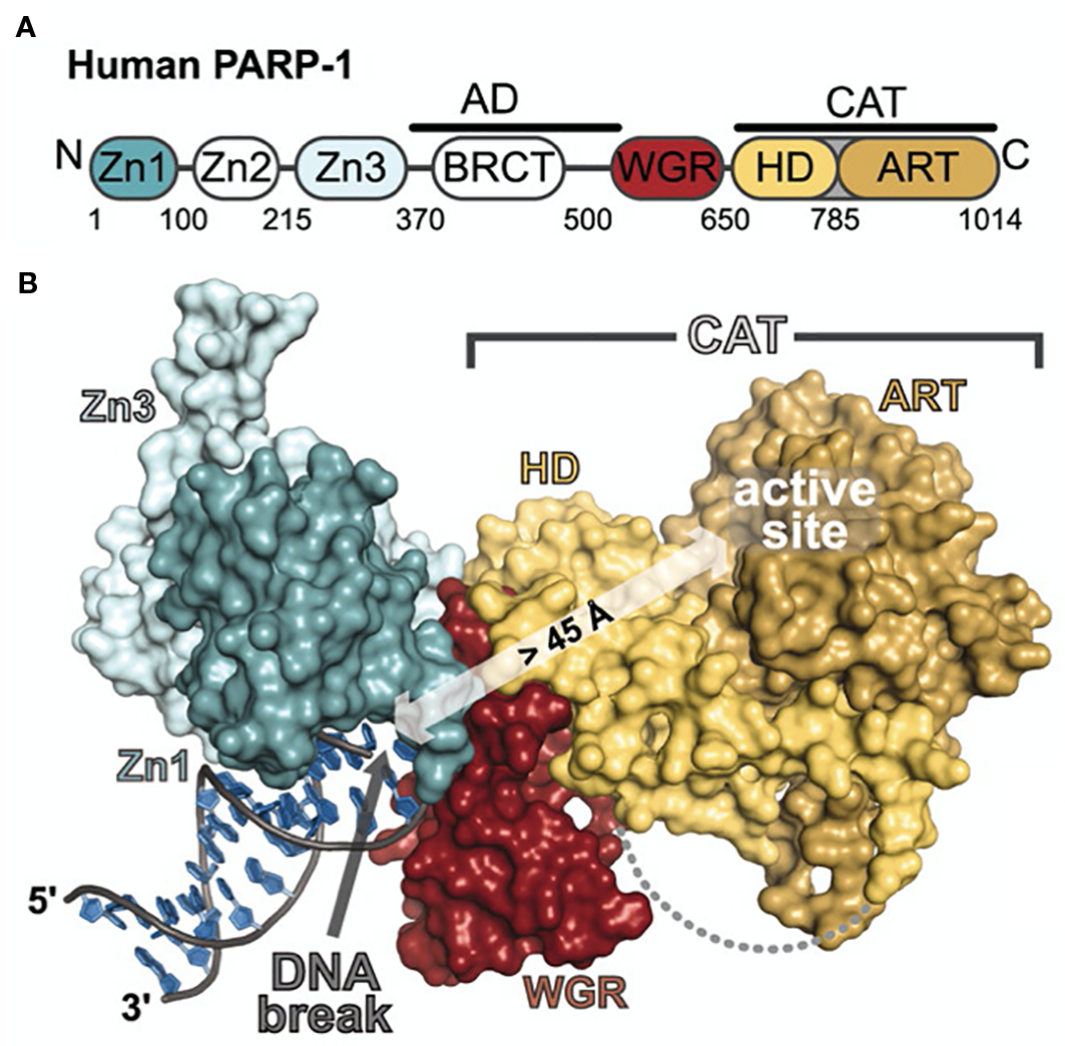
Schematic representation and crystal structure of PARP1 domains and subdomains. **(A)** Schematic representation of human PARP-1 domains and subdomains. A BRCA C-terminus (BRCT) fold is located within the region of PARP-1 that is primarily targeted for automodification. The catalytic domain is composed of an alphahelical subdomain (HD) and an ADP-ribosyl transferase subdomain (ART). **(B)** Crystal structure of the PARP-1/DNA structure. Zn1, Zn3, and WGR-CAT are shown as surfaces. The arrow indicates the location of the PARP-1 automodification region near the catalytic active site. From Langelier et al., 2012. Reprinted with permission from AAAS. [PDB code 4DQY, (Langelier et al., 2012)].

PARP1 is composed of six domains. Moving from the N-terminus to the C-terminus, the enzyme contains three zinc fingers (Zn1, Zn2, Zn3), one domain for auto-poly(ADP-ribose)ylation (autoPARylation; AD) that contains a breast cancer 1 protein (BRCT) motif on the c-terminus of the domain, one domain that interacts with open chromatin (WGR) (Altmeyer et al., 2009; Thomas et al., 2019), and one domain associated with the enzyme’s catalytic activity (CAT) comprised of a helical subdomain (HD) and a conserved ADP-ribosyl transferase subdomain (ART). Zn1 and Zn2 are homologous domains that recognize and bind DNA, though it has been shown that the enzyme can bind DNA with only one of these two domains (Langelier et al., 2011) (**Figure 1**). Taken together, the Zn fingers engage not specific sequences of DNA but rather structural motifs such as blunt ends, single strand breaks (SSBs), double strand breaks (DSBs), 3′ single-base overhangs, and long overhangs (D’Amours et al., 1999; D’Silva et al., 1999; Pion et al., 2003). It is important to note that in each of these cases, PARP1 binds to the irregular angle in the broken DNA strand, not the exposed nucleotides (Lonskaya et al., 2005). Furthermore, each Zn finger seems to play a particular role in a different aspect of the enzyme’s function. For example, Zn1 is responsible for binding DSBs, interacting with the catalytic domain, and activating PARP1. Zn2 seems to be responsible for the recognition of SSBs. (Eustermann et al., 2011). Zn3 has been shown to play a critical role in protein–protein interactions during DNA-dependent autoPARylation by initiating hydrolysis of the NAD^+^ substrate (Langelier et al., 2008; Venere et al., 2014). Unlike Zn1 and Zn2, Zn3 is not required for DNA activation, though it does mediate PARP1–chromatin interactions (Langelier et al., 2010; Venere et al., 2014).

### Single and Double Strand DNA Repair

The complete mechanisms of poly(ADP-ribosyl)ation by the PARP family of enzymes and its role in DNA repair and regulation warrants further investigation (Alemasova and Lavrik, 2019). One proposed hypothesis is the homodimerization of PARP1 after recruitment to the side of DNA damage to begin autoPARylation and the repair process (Bauer et al., 1986; Bauer et al., 1990; Mendoza-Alvarez and Alvarez-Gonzalez, 1993; Mendoza-Alvarez and Alvarez-Gonzalez, 1999; Mendoza-Alvarez and Alvarez-Gonzalez, 2004). PARP1 has been shown to dimerize in its active form in solution which, greatly increased its specific activity (pmol of product/min per pmol of enzyme) (Bauer et al., 1990). PARP1 dimerization was also confirmed by dynamic light scattering (Vasil’eva et al., 2019). The rate of automodification is a function of PARP concentration consistent with second order kinetics. The rate kinetics suggest a dimerized model in which two PARP1 enzymes initialize PARylation, each with an active NAD^+^ binding site (Mendoza-Alvarez and Alvarez-Gonzalez, 1993; Mendoza-Alvarez and Alvarez-Gonzalez, 1999; Mendoza-Alvarez and Alvarez-Gonzalez, 2004). Interestingly, an earlier study supports a model where optimal enzyme activity and PAR chain formation occur in a PARP1:DNA binding stoichiometry of 2:1 where one PARP1 enzyme is catalytically active and the other PARP1 acts as a receptor for PARylation (Panzeter and Althaus, 1994). This model is supported by the crystal structure of the Zn1 and Zn2 domains from separate PAPR1 enzymes in a dimer, complexing damaged DNA (Ali et al., 2012). Another study found the 5′-recessed structure of DNA will recruit PARP1 in a 2:1 PARP1:DNA stoichiometry (Pion et al., 2003). Once dimerized, PARylation and autoPARylation are initiated. AutoPARylation takes place on glutamate and lysine residues found in the AD (Altmeyer et al., 2009; Venere et al., 2014). This domain is also the site of protein–protein interactions (WGR) with the downstream protein targets of PARP1 (Venere et al., 2014). However, in the absence of an AD domain, PARP1 can still PARylate other proteins (Altmeyer et al., 2009).

There are several proteins which can be modified with potentially large, negatively charged PAR chains, which have been found to be between 1 and 200 units long (Hakmé et al., 2008). It has been suggested that as the length of the PAR chain increases the affinity of PARP1 for DNA decreases, mostly due to its highly negative charge, allowing PARP1 to release the DNA and leave space for other DNA repair proteins to bind (Ogata et al., 1980; Poirier et al., 1982; Tulin and Spradling, 2003; Timinszky et al., 2009). The PAR chain can be hydrolyzed to shorter PAR chains, mono(ADP-ribose) by ADP-ribose hydrolase (ARH3) or PAR glycohydrolase (PARG) (Oka et al., 2006; Min and Wang, 2009). When ARH3 or PARG cleaves the first ADP-ribose in a PAR chain from PARP it reestablishes the enzyme’s ability to recognize and bind DNA damage, essentially “resetting” the PARP (Rouleau et al., 2010).

The most direct use of the PAR chain is as energy in the form of ATP when the cell is experiencing decreased levels of ATP (Petermann, 2003; Walker et al., 2006). It is known that DNA damage leads to rapid depletion of ATP reserves in the cell (Sims et al., 1983). This lends to the hypothesis that one role of PARP is to provide ATP from NAD^+^ for the ligation step in the BER pathway, which is favored in times of ATP depletion (Petermann, 2003; Walker et al., 2006).

The PAR chain has been reported to have the potential to recruit an array of different proteins (Rouleau et al., 2010). When interacting with acceptor proteins, the PAR chains can modulate localization, function, and structure (Kraus, 2008; Krishnakumar and Kraus, 2010). Aside from autoPARylation, target proteins for PARylation by PARP1 include other DNA repair proteins, transcription factors, histones, and chromatin modulators (D’Amours et al., 1999; Hassa and Hottiger, 2008). One of the PARylation targets during DNA damage repair is H1, whose targeting results in the relaxation of the chromatin super structure and recruitment of repair proteins such as XRCC1 (El-Khamisy, 2003; Okano et al., 2003). XRCC1 binds directly to the PAR chain, whereas other repair proteins interact with mediating proteins that in turn bind PAR (Rouleau et al., 2010).

Necessary, additional interactions between DNA repair proteins occur through a BRCA1 carboxy-terminal (BRCT) repeat motif found in many other proteins involved in DNA damage repair (Kameshita et al., 1984). This domain, containing a conserved ADP binding sequence comprised of 20 amino acids, has been identified and found to overlap with domains in many proteins associated with DNA binding, nuclear localization, nuclear export, protein degradation, and protein–protein interaction (Pleschke et al., 2000). Two enzymes essential to HR, ataxia telangiectasia-mutated (ATM) and mitotic recombination 11 (MRE11) are signaled through PAR as well (Haince et al., 2008; Sugimura et al., 2008).

### Synthetic Lethality

Exploiting synthetic lethality—a relationship between two cellular mechanisms wherein the functional loss of one is survivable but the loss of both is lethal—was proposed as a treatment for cancer almost a quarter of a century ago (Hartwell et al., 1997). This strategy was validated during the phase I trial of the PARP1/2 inhibitor olaparib, when the majority of patients with BRCA1/2 mutations saw a benefit from PARPi intervention (Fong et al., 2009). In 2014, olaparib received FDA approval for treatment of advanced ovarian cancer (Kim et al., 2015).

When the homologous recombination (HR) pathway is compromised, inhibition of the remaining PARP-dependent BER pathway can be lethal to cells, although the exact mechanism is not entirely understood (Helleday, 2011). Presently, the model for PARPi-mediated synthetic lethality is that an inhibitor will bind a DNA-bound PARP enzyme, preventing it from PARylating proteins or dissociating from the DNA. These lesions, caused by trapped PARPs, accumulate and prevent DNA repair and replication, causing cytotoxicity (Murai et al., 2012; Murai et al., 2014; Pommier et al., 2016). Likely, the HR pathway fails in BRCA-deficient cells due to loss of function of BRCA1/2, causing the cells to rely upon BER to repair damaged DNA (Ström et al., 2011). The BRCA1 and BRCA2 genes code for the eponymous tumor-suppressing proteins essential to the HR repair pathway (Roy et al., 2011). Loss of function of one of these genes *via* mutation is associated with a high risk of breast and ovarian cancer (Miki et al., 1994; Wooster et al., 1995). These types of BRCA1/2 negative cancers are naturally sensitive to PARP inhibitors (Bryant et al., 2005; Farmer et al., 2005; Liu et al., 2008; Rottenberg et al., 2008; Fong et al., 2009; Evers et al., 2010).

While synthetic lethality *via* PARP inhibitors is frequently associated with BRCA1/2 mutations in the literature, many genes play crucial roles in various stages of the HR repair pathway (Cejka, 2015; Hoa et al., 2015). An example of how PARP inhibitors can be lethal to HR-deficient cells is their effect on XRCC1-deficient cells. An increased amount of SSBs were detected when XRCC1-deficient cells were treated with a PARP inhibitor (Ström et al., 2011). Certain tumors arising from hereditary cancers that share an HR-deficient phenotype, not just BRCA deficiency, are sometimes described by the term “BRCAness.” This includes any mutation that would affect replication fork stability, or any genes involved in the HR pathway, for example ATM, ATR, FANC, or PALB2 (Turner et al., 2004; McCabe et al., 2006; Lord and Ashworth, 2016). There is evidence of BRCAness and PARP inhibitors inducing synthetic lethality in cancers that are known to have HR-deficient pathways, such as high-grade serous ovarian cancer (HGS-OVCa), advanced prostate cancer, and pancreatic cancers (Bell et al., 2011; Carnevale and Ashworth, 2015; Mateo et al., 2015; Waddell et al., 2015; Lord and Ashworth, 2016).

The first evidence of PARP inhibition inducing lethality appeared in the 1970s when NAD^+^ analogs were used to bind PARP1 in combination with a genotoxic agent (Brightwell et al., 1975; Terada et al., 1979; Purnell and Whish, 1980). At the time of writing, four therapeutic PARP inhibitors have been approved by the FDA (olaparib, rucaparib, niraparib, and talazoparib) and four more are in various stages of clinical trials (veliparib, E7016, CEP-9722, BGB-290; NCT01827384, NCT01605162, NCT01345357, NCT03150810, respectively).

### Increased Genomic Instability After PARP Inhibition

Originally, the mechanism proposed for PARP-inhibitor-mediated synthetic lethality was the accumulation of DSBs produced when a replication fork failed after encountering an inhibited PARP bound to an SSB (Farmer et al., 2005). There is evidence that cells undergoing PARP inhibition contain no significant increase in SSBs (Gottipati et al., 2010; Ström et al., 2011). PARP knockout cells and PARP knockdown cells contain no higher level of SSBs than wildtype cells (Fisher et al., 2007). These findings suggest alternate explanation of PARP inhibitor-mediated synthetic lethality.

PARP more directly affects the genome through PARylating histones and other nuclear proteins to unwind the chromatin structure (De Murcia et al., 1986; Althaus et al., 1994). An increased level of biomarkers of genomic instability, such as DNA strand breaks, gene amplification, DNA recombination, and SCE were found in cells with decreased PARP activity after treatment with DNA-damaging agents. These findings were made using PARP inhibitors, PARP knockout models, and asRNA models (Küpper et al., 1990; Waldman and Waldman, 1991; Ding and Smulson, 1994; Schreiber et al., 1995).

These results lead to the hypothesis that when HR and BER pathways are inaccessible to cells, they rely on non-homologous end joining (NHEJ). HR and BER are conservative DNA repair methods, maintaining the original DNA sequence that was damaged. When HR is not an option for the cell, it has to rely on BER and NHEJ. NHEJ is a non-conservative repair pathway because it will excise the damaged DNA, leading to genomic instability (Moynahan et al., 2001; Tutt et al., 2001). It has been shown that NHEJ is promoted in cells with defective HR pathways after treatment of PARP inhibitors. Also, resistance to PARP inhibitors is acquired when the NHEJ pathway is inhibited (Patel et al., 2011). These findings indicate that PARP-inhibitor-induced lethality can also be attributed to genomic instability as a result of the NHEJ pathway in non-irradiated situations.

### PARP Trapping

PARP trapping is the formation of a PARP–DNA complex through inhibition of DNA-bound PARP. PARP–DNA complexes were detected in cell lines treated with olaparib and rucaparib (Murai et al., 2012; Murai et al., 2014). PARP inhibitors prevent PARP from synthesizing PAR chains by competitively binding PARP’s natural substrate, NAD^+^.

Interestingly, inhibiting PARP is more cytotoxic than the absence of PARP itself (Thomas et al., 2018). One hypothesis for this effect might be due to replication fork stalling and subsequent collapse, a mechanism shared with topoisomerase I (TOP1) and TOP1 inhibitors. Further evidence of PARP trapping and collapsing replication forks is PARP1’s role in restarting stalled replication forks, a task prevented by PARP inhibitors (Yang et al., 2004; Bryant et al., 2009). This mechanism sheds some light on the lethality of PARP inhibitors in cells with and without BRCA mutations (Strumberg et al., 2000).

The efficacy of PARP trapping was shown to be independent of the half-maximal inhibitory concentration (IC_50_) of the PARP inhibitors (Murai et al., 2012; Murai et al., 2014). Of the FDA-approved PARP inhibitors, veliparib is the least effective at PARP trapping, irrespective of the fact that its IC_50_ value is lower than that of niraparib (2 nM and 3.2 nM, respectively). Olaparib has a significantly more favorable IC_50_ than rucaparib (5 nM and 1.4 nM for olaparib and rucaparib, respectively), and they exhibit equal efficacy as PARP trapping agents. Talazoparib has the more favorable IC_50_ and functions as the best PARP trapping agent (Murai et al., 2012). Talazoparib’s ability to trap PARP is likely due to its bulky structure and rigidly which contributes to a slow rate of dissociation (Shen et al., 2013; Murai et al., 2014; Pommier et al., 2016). Recent combination trials have demonstrated new indications of PARP inhibitors in combination with other therapeutics, extending their use beyond cancers with BRCAness. One such example includes a combination therapy of rucaparib and temozolomide in metastatic melanoma, a cancer not typically associated with BRCA1/2 mutations (Plummer et al., 2013).

### PARP Inhibition as a Radiosensitizer

While PARP inhibition in cancers with “BRCAness” can induce synthetic lethality, PARPi in other cell lines can radiosensitize them. The PARPi 3-aminobenzamide was able to radiosensitize two breast cancer cell lines, MDA-MB-231 and MDA-MB-436, one with and one without BRCA mutation, respectively (Zhao et al., 2019). The mechanism of PARPi induced radiosensitivity is most likely a DNA replication-dependent model in which replication forks collapse during delayed SSB repair, as demonstrated by Dungey et al. in which a replication-dependent increase in ɣH2AX foci in G2 cells was observed after treatment with olaparib and fractionated ionizing radiation (IR) in the T98G model of glioblastoma (Dungey et al., 2008). Noel et al. showed HeLa cells were radiosensitized by the PARPi 4-amino-1,8-naphthalimide during S phase. Irradiation of these cells produced an increase of DSBs hours after irradiation (Noel et al., 2006). Evidence supporting a cell-cycle dependent effect was provided earlier by Chalmers et al. when hamster fibroblast cell lines V79-379A and CHO-K1 and human glioma cell line T98G treated with PARPis were the most radiosensitive during periods of rapid growth. Once cells had been arrested in G1 phase, radiosensitivity was lost (Chalmers et al., 2004).

## Preclinical Models of Combination Therapies

### *In Vitro*—Clonogenic Assays

The Marples group out of Wayne State University has demonstrated that radiosensitivity can be increased through PARP inhibition in human glioma cell lines U373-MG and T98G. Clonogenicity was evaluated with increasing concentrations of PARP inhibitors (1–3 μM) that were found to be non-toxic in the absence of radiation. A 3 μM concentration of PARP inhibitor, the highest concentration of inhibitor that was non-toxic in the absence of IR, was then used in conjunction with low levels (0.05–0.3 Gy) of ionizing radiation (IR) to induce toxicity (Chalmers et al., 2004). Treatment with a small molecule PARP inhibitor, AG14361, followed by 8 Gy IR reduced survivability in colorectal cancer cell lines (LoVo) by 73% (Calabrese et al., 2004). Non-small cell lung cancer (NSCLC) cell lines A549 and Calu-6 were each treated with 1 μM and 5 μM of olaparib before being exposed to 0, 2, 4, and 6 Gy to find dose-dependent sensitization of both cell lines. For A549 and Calu-6 the Survival Enhancement Ratios (SER) values at 1 μM were found to be 1.3 and 1.5, respectively. These values increased to 1.6 μM and 1.8 at 5 μM (Senra et al., 2011). Veliparib was shown to have a limit on radiosensitization with concentrations above 2.5 μM no longer increasing radiosensitivity in a NSCLC cell line, H1299. Survival fractions were decreased when IR was supplemented by pretreatment of 2.5 μM veliparib. This effect was also observed in human prostate cancer cell lines (DU145 and 22Rv1) (Liu et al., 2008). The PARP inhibitor E7016 was able to increase radiosensitivity across multiple cancer cell lines as well. A dose enhance factor ≥1.4 was calculated for glioblastoma (U251) and pancreatic (MiaPaCa), and prostate cancer (DU145) cell lines when treated with E7016 prior to IR. Surviving fractions in all three cell lines were reduced to 0.1 in clonogenic assays (Russo et al., 2009). Veliparib demonstrated no effect on colony formation in PC-3 prostate cancer cells when incubated in 10 μM veliparib. The same treatment, followed by 2 Gy IR, reduced colony formation to 47% (Barreto-Andrade et al., 2011). 22Rv1 prostate cancer cells had PARylation reduced by 97–100% after incubation with the PARP inhibitor olaparib. The radiosensitization enhancement ratio was found to be ≥1.2 when combined with IR compared to PARP inhibitor alone. This result was found in acutely hypoxic, chronically hypoxic, and normoxic conditions (Gani et al., 2015). These works establish the efficacy of a variety of PARP inhibitors as radiosensitizers for multiple human cancer cell lines at low μM concentrations, often below cytotoxic concentrations of the PARP inhibitors themselves.

### *In Vivo*—Tumor Growth Delay and Survival

Combination therapy is a more efficacious approach to treating H460 models of non-small cell lung cancer. A tumor growth delay assay using a five-fold increase in tumor volume as an endpoint saw a 1-day or 7-day delay using the PARP inhibitor veliparib or external beam radiation alone, respectively. When these therapies were combined, the five-fold increase in tumor volume was delayed by 13.5 days (Albert et al., 2007). A subcutaneous LoVo xenograft model of colorectal cancer exhibited tumor growth delay of 19 days with a fractionated regimen of IR that was increased to 37 days when combined with a low dose of AG14361, which did not delay tumor growth when administered alone (Calabrese et al., 2004). Tumor growth was significantly impeded in a dose-dependent trend of GPI-15427 and 2 Gy in mouse models of JHU006 and JHU012 HNSCC (Khan et al., 2009). A dose response dependency of veliparib was demonstrated in a human colon cancer mouse xenograft model, HCT116, when administered through a subcutaneously implanted osmotic pump in conjunction with IR compared to IR alone (Donawho et al., 2007). Calu-6 mouse xenograft models received a daily 50 mg/kg dose of olaparib for 5 days, 5 days of 2 Gy IR daily, or both therapies. The combination therapy cohort experienced a significant delay of 10 days to reach the endpoint compared to either monotherapy or control cohort (Senra et al., 2011).

Mouse models of HCT116 colorectal cancer receiving twice daily orally administered doses of 12.5 mg/kg of veliparib in conjunction with 2 Gy fractions of IR displayed significant tumor growth delay compared to control groups of IR alone (Shelton et al., 2013). Olaparib was also tested as a radiosensitizer in subcutaneous mouse models of glioblastoma-initiating cells. Daily treatment of olaparib over 7 days was administered concurrently with 3 Gy of IR every other day for three total doses to find greater tumor growth delay than vehicle, IR alone, or IR with vehicle (Venere et al., 2014). The effect of fractionated RT after sensitization by olaparib was evaluated in a 22Rv1 human prostate cancer mouse model. One cohort received a single 8 Gy dose on the third day of three consecutive daily doses of intraperitoneally administered PARP inhibitor. A second cohort was treated with seven consecutive days of olaparib, with 5 × 2 Gy doses every other day starting 3 days after the initial PARP inhibitor injection. The group receiving fractionated doses displaying a non-significant delay in tumor growth compared to the vehicle + fractionated IR control group (Gani et al., 2015). The above data suggests that a combination therapy between PARP inhibitors and RT is more effective *in vitro* and *in vivo* than either therapy alone and is summarized in **Table 1**.

**Table 1:** Preclinical combination therapies with PARP inhibitors and ionizing radiation.

PARPi	Disease	Cell line	Assay	Publication
AG14361	Colorectal	LoVo	Clonogenic	Calabrese et al., 2004
			Tumor growth delay	
E7016	Glioblastoma	U251	Clonogenic	Russo et al., 2009
	Pancreatic	MiaPaCa		
	Prostate	DU145		
GPI-15427	HNSCC	JHU006	Tumor growth delay	Khan et al., 2009
Olaparib	NSCLC	A549	Clonogenic	Senra et al., 2011
		Calu-6		
			Tumor growth delay	
	Glioblastoma	GIC	Tumor growth delay	Venere et al., 2014
	Prostate	22Rv1	Tumor growth delay	Gani et al., 2015
			Clonogenic	
PJ34	Glioblastoma	U373-MG	Clonogenic	Chalmers et al., 2004
		T98G		
Veliparib	NSCLC	H460	Tumor growth delay	Albert et al., 2007
		JHU012		
	Colon	HCT116	Tumor growth delay	Donawho et al., 2007
	NSCLC	H1299	Clonogenic	Liu et al., 2008
	Prostate	DU145		
		22Rv1		
	Prostate	PC-3	Clonogenic	Barreto-Andrade et al., 2011
			Tumor growth delay	
	Colon	HCT116	Tumor growth delay	Shelton et al., 2013

## Combination Therapy Clinical Trials

### Ionizing Radiation With Chemotherapy and PARPis

There are currently several completed clinical trials exploring the efficacy of combining PARP inhibitors, radiotherapy and chemotherapy, none of which have available results. In a phase I study of patients with phase II or III rectal cancer, patients were given 825 mg/m^2^ capecitabine twice daily and 1.8 Gy fractions daily for a total of 50.4 Gy over approximately 6 weeks in conjunction with escalating doses (20–400 mg) of veliparib orally twice daily. Maximum tolerated dose was not reached, and the study found 400 mg twice daily to be the appropriate dose of veliparib for the phase II study (NCT01589419, Czito et al., 2017). Two other phase I studies evaluating the combination of veliparib and temozolomide against diffuse pontine glioma and glioblastoma have also concluded, but results have not yet been posted (NCT01514201, NCT00770471). The PARADIGM-2 study is two parallel phase I studies in which one arm evaluated dose escalation of olaparib (50–150 mg/daily) is combined with 60 Gy in 30 fractions over 3 weeks of radiotherapy followed by four additional weeks of olaparib. The second arm included the same regimen with concomitant temozolomide at 75 mg/m^2^ daily throughout radiotherapy and again after the 4 weeks of olaparib (Fulton et al., 2018). One study aims to find the MTD of b 25–200 mg of olaparib twice daily beginning 3 days prior to the first dose of cetuximab. The initial 400 mg/m² dose of cetuximab will precede the start of radiation by 5–7 days. 69.3 Gy of radiation therapy will be administered in 33 fractions over 6.5 weeks (NCT01758731).

### Ionizing Radiation With PARPis

The first clinical trial exploring combination therapy between PARP inhibitors and ionizing radiation to publish results combines veliparib and whole brain radiation therapy (WBRT) in adult patients with brain metastases from non-small cell lung cancer (NCT00649207). Patients were age >18 years with Karnofsky performance status (KPS) scores ≥70. One arm received WBRT administered daily in 2.5 Gy fractions over 15 sessions for 37.5 Gy total. A second arm was treated with 150 mg of veliparib twice daily with concurrent daily fractions of 3.0 Gy fractions over 2 weeks for 30 Gy.

All three arms of the study received a 30 Gy fractionated dose of 10 × 3 Gy doses, excluding weekends and holidays. The variable was the quantity of drug received twice daily: placebo, 50 mg veliparib, or 200 mg veliparib. The primary outcome was survival up to 36 months. While the patient tumor population was homogenous, 88–90% of patients in this trial had Graded Prognosis Assessment scores ≤2.5, amounting to an unfavorable prognosis, and the primary outcome was not met (Chabot et al., 2017). It is worth noting that even when a combination therapy significantly prolongs survival in patient populations with favorable prognoses (GPA 2.5–4), it falls short of significance in populations with unfavorable prognoses (Aoyama et al., 2015). This study progressed to phase II (NCT01657799), where no benefit was found in combining WBRT with veliparib compared to WBRT and a placebo (Chabot et al., 2017).

## New Frontiers—PARPi Diagnostics and Radiotherapies

### PARPi-FL

PARPi-FL was first reported in 2012 by the Weissleder Lab at Massachusetts General Hospital in human pancreatic cancer cells (Reiner et al., 2012). It can be used for real-time visualization of intracellular kinetics of PARP inhibitors (Thurber et al., 2013). It was later shown to be a viable imaging agent *in vivo* in a mouse model of glioblastoma (Irwin et al., 2014). Composed of a BODIPY-FL dye conjugated to an olaparib scaffold, it retains a similar pharmacokinetic profile, including the low IC_50_ value of 12.2 nM compared to the 5.0 nM value of olaparib (Menear et al., 2008). It can also be blocked by pretreatment with olaparib. PARPi-FL uptake in tumors is rapid, with statistically significantly increased tumor-to-muscle and tumor-to-brain ratios of ≥10 in a mouse model of glioblastoma. Uptake of PARPi-FL was correlated to PARP1 expression, and increased after irradiation (Irwin et al., 2014; Kossatz et al., 2016). Retention persists for hours, with <50% metabolites present in the blood at peak uptake in tumors (Irwin et al., 2014). PARPi-FL has been used for real-time measurements of drug–target interaction *in vitro* and *in vivo* (Dubach et al., 2014; Dubach et al., 2017). The translational potential of PARPi-FL was highlighted when high tumor-to-organ ratios were observed in an orthotopic model of oral squamous cell carcinoma using clinical imaging instruments (Kossatz et al., 2016; Carney et al., 2017), and early clinical outcomes have been reported [NCT03085147 and (Kossatz et al., 2019)].

### PARPi-Derived PET Tracers

**Figure 2:**
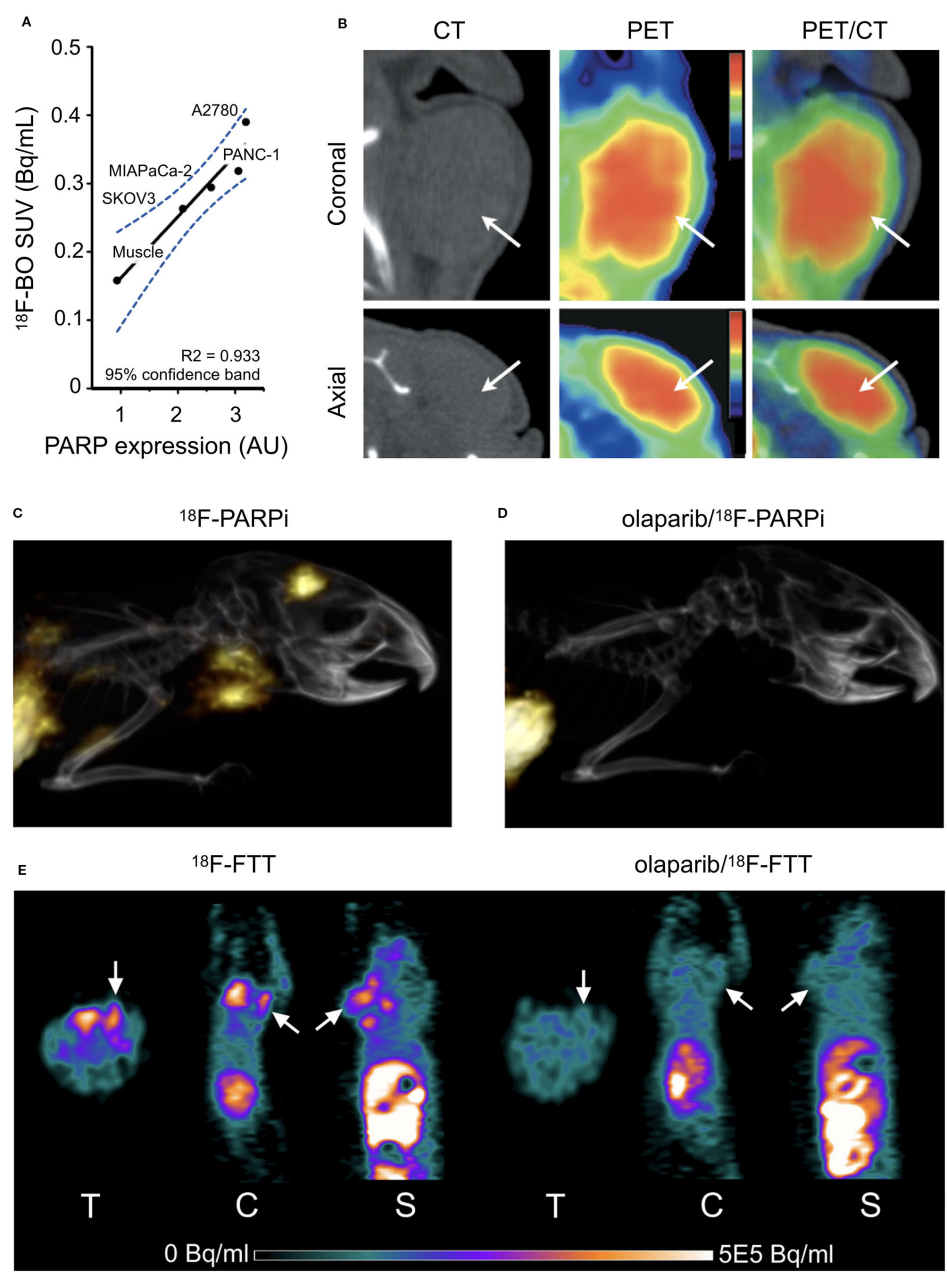
*In vivo* PET/CT Imaging. **(A)** Correlation of ^18^F-BO uptake and PARP expression in two ovarian and two pancreatic tumor types as determined by immunoblotting. **(B)** Coronal and axial PET/CT scans of a subcutaneous A2780 tumor-bearing mouse (Reiner et al., 2012). **(C)** Sagittal PET/CT images of orthotopic U251 MG tumor-bearing mice 2 h post-injection of ^18^F-PARPi. **(D)** PET/CT 2 h post-injection ^18^F-PARPi after 30 m pre-injection of 500-fold excess olaparib (Carney et al., 2016). **(E) **MicroPET images of MDA-MB-231 tumors in mice tumor at 60 min after ^18^F-FTT injection before and after treatment with olaparib (ip 50 mg/kg 20 min pretreatment; Zhou et al., 2014). Reprinted by permission from Springer Nature: Springer Molecular Imaging and Biology (Carney et al., 2016) (Non-invasive PET imaging of PARP1 expression in glioblastoma models, Carney, B, G Carlucci, B Salinas, V Di Gialleonardo, S Kossatz, A Vansteene, VA Longo, A Bolaender, G Chiosis, KR Keshari, WA Weber, and T Reiner), copyright (2016).

The first radiolabeled PARP inhibitor for PET imaging was designed to monitor tissue necrosis. The Mach Group at Washington University labeled the small molecule PARP1 inhibitor PJ34 with carbon-11 and had good yields with increased uptake in target tissue in a rat model of Type I diabetes (Tu et al., 2005). The first reported fluorine-18-labeled PARP inhibitor was [^18^F]FE-LS-75 from the Roesch Group at Johannes Gutenberg-University, which showed high yields up to 80% (Riss et al., 2009) but did not report *in vitro/vivo* experiments. The first fluorinated PARP-targeted small molecule based on a later FDA-approved PARP inhibitor was ^18^F-BO (Keliher et al., 2011). Uptake was shown to correlate to PARP1 expression in breast cancer mouse models. A dose of olaparib prior to injection with ^18^F-BO was able to reduce uptake *in vivo*. Favorable uptake was also observed in pancreatic and ovarian cancer models (Reiner et al., 2012) (**Figures 2A, B**). ^18^F-PARPi-FL was developed as a dual modality PET/fluorescent imaging agent (Keliher et al., 2014). PARP1-specific uptake was demonstrated in glioblastoma xenografts. Both modalities showed similar tumor-to-brain uptake ratios (PET, 9:1; fluorescence, 7:1) as determined by autoradiography and fluorescence microscopy (Carlucci et al., 2015). ^18^F-PARPi is an olaparib-based PET imaging agent that exhibits high specificity for PARP1 in mouse models of orthotopic glioblastoma, diffuse intrinsic pontine glioma, and small-cell lung cancer (Carney et al., 2016; Kossatz et al., 2017; Carney et al., 2018) (**Figures 2C, D**). ^18^F-PARPi has potential to non-invasively monitor disease progression and is currently in phase I clinical trials [(Schöder et al., 2019), NCT03631017]. Wilson et al. has published the synthesis and *in vivo* biodistribution of a fluorine-18 isotopologue of olaparib. Pre-irradiation of the cells and tumors was shown to increase uptake of the compound in several pancreatic cancer cell lines (Wilson et al., 2019). ^18^F-FluorThanatrace (^18^F-FTT) is a rucaparib-based PET imaging agent first published in a human breast cancer mode (Zhou et al., 2014) (**Figure 2E**). It was the first PARP-targeted PET imaging agent to be tested in the clinic and is currently involved in several phase I clinical trials, evaluating uptake in different cancers (Michel et al., 2017; Makvandi et al., 2018).

### PARPi Radiotherapeutics

**Figure 3:**
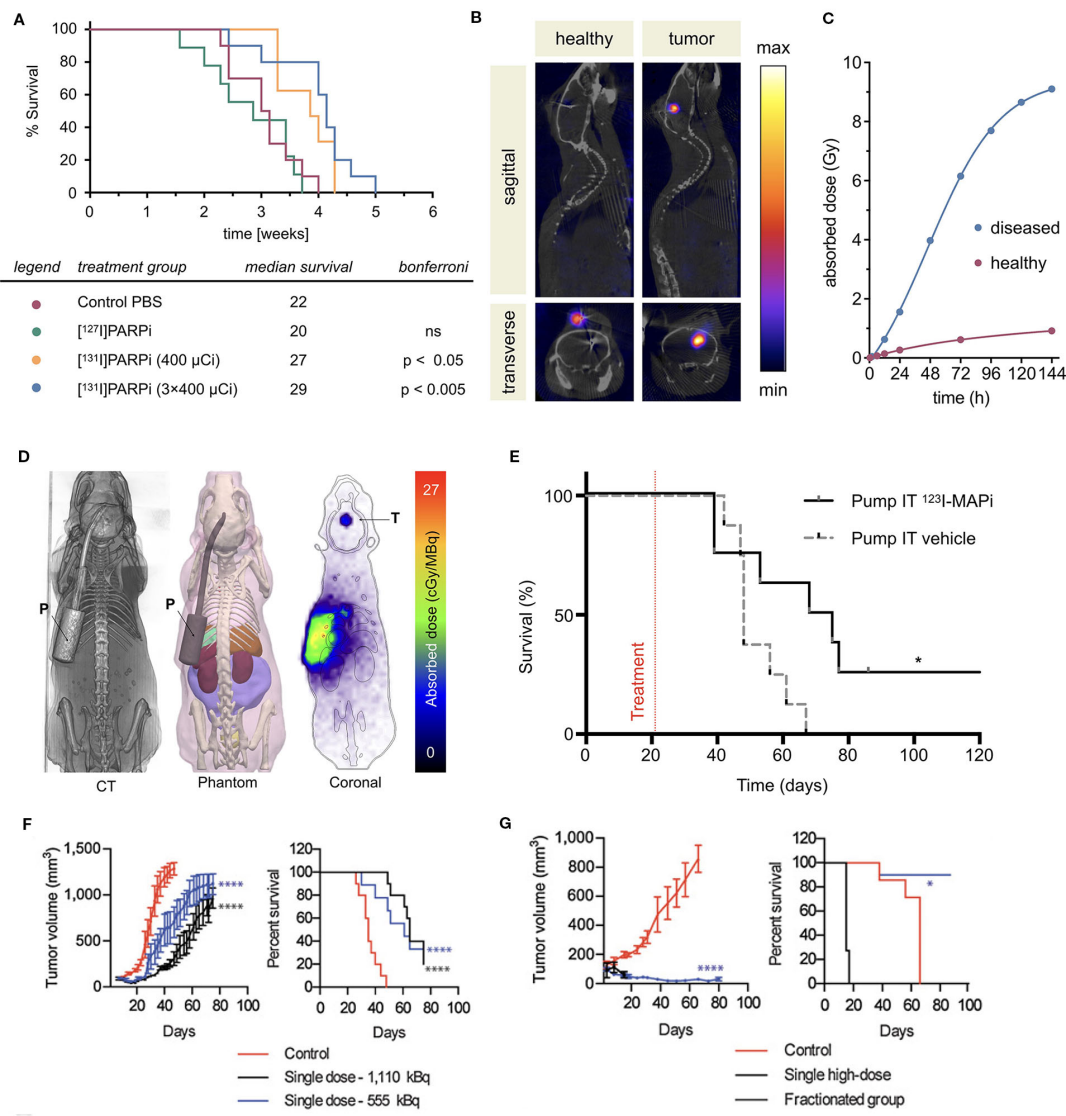
PARP targeted radiotherapies. Radiolabeled PARP inhibitors in mouse models of cancer. **(A)** Survival plot and table of treatment groups with median survival of subcutaneous U87-p53 tumor-bearing mice. P = 0.0001. **(B)** SPECT/CT of orthotopic U87-p53 tumors during osmotic pump treatment of [^131^I]PARPi at 72 hours. **(C)** Calculated absorbed dose to brain* during treatment. *Brain and tumor have been considered together as a single organ in organ level dose calculation. (Jannetti et al., 2018). **(D)** Dosimetry of the subcutaneous pump model showing CT, phantom, and Monte Carlo simulation of dose accumulation in the tumor. **(E)** Kaplan–Meier survival study of pump implanted mice shows an improvement of survival of ^123^I-MAPi treated mice (n = 8) when compared to control (n = 8). *P < 0.05 (Pirovano et al., 2019). **(F)** Tumor growth and Kaplan–Meier curves for IMR-05 tumor**-**bearing mice **(F)** treated with single dose of 555 or 1,110 kBq of [^211^At]MM4 (control *vs*. 555 kBq and 1,110 kBq mixed linear model p < 0.0001; control *vs*. 555 and 1,110 kBq survival Mantel**–**Cox test p < 0.0001, 555 vs. 1,110 kBq not significant, **(G)** and single high dose of 1,480 kBq *vs*. a fractionated dose of 370 kBq twice weekly for a cumulative dose of 1,480 kBq (control *vs*. fractionated mixed linear model p < 0.0001, fractionated *vs*. high dose not significant; survival Mantel**–**Cox test high dose *vs*. control p < 0.0001, fractionated *vs*. control p < 0.03 (Makvandi et al., 2019).

Several PARP inhibitors have also been labeled with therapeutic isotopes (Salinas et al., 2015; Jannetti et al., 2018; Reilly et al., 2018; Makvandi et al., 2019; Pirovano et al., 2019). The pharmacokinetic profiles of several iodinated PARP inhibitors based on olaparib were explored in human glioblastoma models *in vitro* and *in vivo*. Various length linkers were evaluated using an olaparib scaffold and a small library of iodobenzoic acids. The compounds with the best pharmacokinetics were radioiodinated and evaluated in culture and orthotopic mouse models of human glioblastoma for PARP1 specificity (Salinas et al., 2015). The rucaparib scaffold was also leveraged in the design and synthesis of alpha- and auger-emitting radiotherapeutics using copper-catalyzed halogenation of boronic esters (Reilly et al., 2018; Makvandi et al., 2019). The efficacy of PARP-targeted radiotherapeutics was first published in subcutaneous mouse models of glioblastoma, and later in orthotopic models of human glioblastoma (Jannetti et al., 2018; Pirovano et al., 2019). Intratumoral injections were implemented to mimic Convection Enhanced Delivery (CED). A reporter cell line transduced from U87 cells was designed to respond to p53 activation, as well as cellular density. This allowed imaging of cell death following treatment with the iodinated PARP inhibitor. Therapeutic efficacy was evaluated in a tumor growth delay experiment that found a fractionated dose of the drug could significantly delay the endpoint of the study (**Figure 3A**). Use of a CED-mimicking subcutaneous implant allowed approximately 9 Gy to be delivered to a brain-tumor-bearing mouse compared to 1 Gy in a healthy mouse (Jannetti et al., 2018) (**Figures 3B, C**). These results were leveraged in the synthesis and validation of an Auger-electron emitting isotopologue. Uptake of the drug can be decreased by pretreatment with olaparib and proved lethal to cells at concentrations lower than that of olaparib (EC_50_ = 69 nm). The radiotheranostic proved efficacious in prolonging survival of treated mice, and intratumoral administration of the drug in mice bearing human brain tumor significantly increased survival compared to vehicle alone (p = 0.0094). Application of CED-mimicking implants replicated this effect in the same model (p = 0.0361, Pirovano et al., 2019) (**Figures 3D, E**). High radiochemical yields (≥89%) were reported for halogenation of both olaparib and rucaparib scaffolds with astatine-211 and iodine-125 (Reilly et al., 2018). Antitumor effects were observed using an astatinated PARP inhibitor in a mouse model of neuroblastoma. Favorable uptake was observed in the tumor after 2 h. A single dose of the alpha-emitting drug was able to significantly delay tumor growth and prolong survival against a control group (Makvandi et al., 2019) (**Figures 3F, G**).

## Summary and Outlook

Since its discovery we have elucidated the multiple roles PARP1 plays in the cell. PARP inhibitors have given rise to promising new cancer therapies and treatment strategies. We have recently witnessed PARPis receive approval as monotherapies for several cancers, and are waiting on the next generation of these small molecules. Many research groups are already evaluating the potential of PARPis as radiosensitizing agents in preclinical models of combination therapies. PARPis are currently being applied in the clinic as radiosensitizing agents in addition to clinical trials using combination PARPis with chemotherapies and radiation. A handful of these small molecules have been labeled to create a new class of diagnostic and radiotherapeutic agents, several of which are currently in clinical trials. The broad versatility and applications of these PARPis are providing the research community with a new set of tools for diagnosis, patient stratification, and therapy in some of the most lethal cancers.

## Author Contributions

All authors have read and approved the manuscript, and the authors disclose no conflict of interests. The contributions of said authors can be broken down as follows: Drafting the manuscript, or critically contributing to or revising the manuscript, or enhancing its intellectual content: SJ, BZ, MZ, TR. Approving the final content of the manuscript: SJ, BZ, MZ, TR.

## Conflict of Interest

SP, CB, JSL, and TR are shareholders of Summit Biomedical Imaging, LLC. SP and TR are co-inventors on filed U.S. patent (WO2016164771) that covers methods of use for PARPi-FL. TR is a co-inventor on U.S. patents (WO2012074840 and WO2016033293), covering the compositions of matter for PARPi-FL and 18F-PARPi, respectively. TR is a paid consultant for and has received grant support from Theragnostics, Inc., which has licensed 18F-PARPi. This arrangement has been reviewed and approved by Memorial Sloan Kettering Cancer Center in accordance with its conflict of interest policies.
